# Life Threatening Idiopathic Recurrent Angioedema Responding to Cannabis

**DOI:** 10.1155/2015/780824

**Published:** 2015-07-16

**Authors:** Amit Frenkel, Aviel Roy-Shapira, Brotfain Evgeni, Koyfman Leonid, Abraham Borer, Moti Klein

**Affiliations:** ^1^General Intensive Care Unit, Soroka University Medical Center and the Faculty of Health Sciences, Ben-Gurion University of the Negev, 84101 Beersheba, Israel; ^2^Infection Control and Hospital Epidemiology Unit, Soroka University Medical Center and the Faculty of Health Sciences, Ben-Gurion University of the Negev, 84101 Beersheba, Israel

## Abstract

We present a case of a 27-year-old man with recurrent episodes of angioedema since he was 19, who responded well to treatment with medical grade cannabis. Initially, he responded to steroids and antihistamines, but several attempts to withdraw treatment resulted in recurrence. In the last few months before prescribing cannabis, the frequency and severity of the attacks worsened and included several presyncope events, associated with scrotal and neck swelling. No predisposing factors were identified, and extensive workup was negative. The patient reported that he was periodically using cannabis socially and that during these periods he was free of attacks. Recent data suggest that cannabis derivatives are involved in the control of mast cell activation. Consequently, we decided to try a course of inhaled cannabis as modulators of immune cell functions. The use of inhaled cannabis resulted in a complete response, and he has been free of symptoms for 2 years. An attempt to withhold the inhaled cannabis led to a recurrent attack within a week, and resuming cannabis maintained the remission, suggesting a cause and effect relationship.

## 1. Introduction

Angioedema is a potentially life threatening condition which has numerous hereditary, acquired, and iatrogenic causes. Although most patients present with urticaria [1], severe attacks may lead to airway obstruction and even death [2]. There are two main types of angioedema: histaminergic angioedema versus nonhistaminergic angioedema. Both types are equally dangerous and require an aggressive approach to diagnosis and treatment [2].

In most cases of angioedema, careful workup usually identifies the etiology of the attacks and allows appropriate preventive measures. According to guidelines published by The World Allergy Organization (WAO) [3], the workup of patients presenting with signs and symptoms of angioedema (urticaria, flushing, generalized pruritus, bronchospasm, throat tightness, and/or hypotension) includes detailed history of exposure to potential allergens, such as particular food items, drugs, inhaled materials, latex, and stinging insect.

In patients with recurrent episodes, further workup may require additional workup, such as immune components level, thyroid function and antibodies, chest and abdominal imaging, skin and gastrointestinal biopsies, stool examination for parasites, and bone marrow aspiration. The value of aeroallergen screening for patients with angioedema is limited [3].

Idiopathic angioedema (IAE) is defined as recurrent episodes of angioedema without urticaria for which no explanation can be found after full evaluation [4]. Currently recommended therapy is largely empiric. Available treatment modalities include a trial of nonsedating antihistamines, combination of nonsedating antihistamines with a leukotriene receptor antagonist, systemic corticosteroids, and immunosuppressant therapies. Plasmapheresis or intravenous immunoglobulin has also been used [5]. None of these treatments is universally successful.

Recent studies have demonstrated that cannabis has an important role in modulating the immune response [6]. There are at least two types of cannabinoid receptors, CB1 (CNR1) and CB2 (CNR2), both coupled to G proteins. CB2 receptors, the nonpsychoactive cannabinoid receptors, are present mainly on immune cells [7], suggesting that cannabinoids may have an important role in the regulation of the inflammatory response. This suggestion is supported by the observation that cannabinoids effectively suppress immunologic and inflammatory functions of leukocytes in vitro [8] and the reported effect of cannabis in modulating a variety of immune cell functions such as T helper cell development, chemotaxis, and tumor development [9].

Consequently, we hypothesized that the use of cannabis may be effective in the management of patients with refractory IAE.

## 2. Case Presentation

The patient was a 27-year-old man with a history of recurrent episodes of angioedema. The episodes begun when he was 19 years old, and their frequency and severity had been rising over the previous six months. There was a history of urticaria following penicillin administration in early childhood, but there were no other known allergies.

Initially, the episodes were manifested by mild urticaria which resolved spontaneously. But the severity and frequency of attacks increased over the years. In the last few months, the attacks became more frequent, at least twice a month, and included autonomic dysfunction: one episode of syncope and a few presyncopal episodes associated with scrotal and neck swelling and abdominal pain. Two of the episodes were considered “life threatening” due to imminent airway obstruction and were treated with subcutaneous epinephrine and intravenous steroids, which were tapered down gradually after each episode. Due to the severity of the episodes, the patient was instructed to carry epinephrine 0.3 mg autoinjector (EpiPen) at all times.

Extensive search for possible etiology was carried out. Ancillary studies were nonproductive. Blood tests included a complete blood count, full chemistry panel, serum levels of thyroid stimulating hormone, antinuclear antibodies, rheumatoid factor, antineutrophil cytoplasmic antibodies, C-1 inhibitor activity, tryptase, and chromogranin. Urinary vanillylmandelic acid and 5-hydroxyindoleacetic acid were also negative, as were urine and stool tests for OVA and parasites. Imaging studies (chest roentgenograms, abdominal ultrasound) did not show evidence of echinococcal infection, and endoscopically obtained biopsies from the GI tract did not show eosinophils or mast cells. Skin biopsy was negative for C-KIT, and bone marrow aspiration showed no evidence of increased mast cell count and was negative for tryptase and C-KIT. While the workup was in progress, the patient was placed on prophylactic antihistamines, using several preparations, but the attacks persisted, and periodically supplemental steroids were necessary.

The patient disclosed that from time to time he was using marijuana socially and that it seemed to him that the attacks were less frequent when he did. The patient declared that he did not prepare the cigarette himself, so we presumed he did not have a significant skin contact with cannabis. The history was consistent with the known effect of cannabis on the immune system as described in the introduction, so we conjectured that marijuana may have had a modulatory effect facing failure of other treatment modalities. We suggested that the patient should try medical grade marijuana inhalations (cannabis products for medical indications may be legally used in Israel, but authorization by the Ministry of Health is required for each individual patient and prescription must be renewed monthly). The patient was informed that there was no supporting evidence in the medical literature for this treatment in his condition but that the potential risk for side effects was minimal and he consented to try. He was instructed not to drive or engage in other potentially risky activities during treatment.

**Figure 1:**
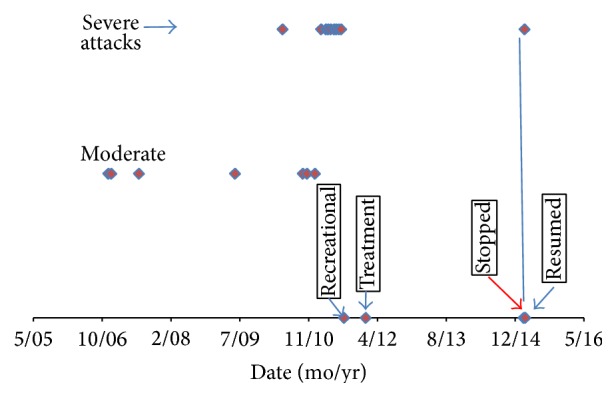
Attack severity by date.

We prescribe a total of 20 grams of medical grade marijuana per month, inhaled two to three times a week, which in Israel is the minimal accepted dosage for medical indications. The patient stayed on this dose for two years during which the patient did not have a single attack and no adverse effects were observed. After two years, the patient tried to stop treatment, on his own initiative, but developed a recurrent severe attack a week later. After resuming the treatment no further attacks were observed over the next 4 months of close followup (the regulations for legal use of medical marijuana stipulate that the prescription must be renewed monthly). The timeline of the course of the disease is depicted in Figure 1.

## 3. Discussion

This is the first report in which a cannabis product for the treatment of refractory idiopathic angioedema was associated with an excellent clinical response. The rate of attacks dropped from at least twice a month to nil, appeared again a week after cessation, and disappeared again when treatment was restarted. This course is consistent with Koch's postulates for establishing causality: frequent attacks before treatment, no attacks during treatment, and recurrence after temporarily stopping treatment.

The exact mechanism by which the drug worked is not clear. Measuring IgE against LPTs (e.g., Pru p3) [7] may have shed some light on the mechanism because cross reactivity with cannabis has been described, but this test is not currently available in our institute.

In this case, the patient smoked medical grade marijuana, which affects both the CB1 and CB2 receptors. The CB2 cannabinoid receptors are present mainly in immune cells [6]. It is possible that using CB1 antagonists would allow elimination of potentially undesirable psychotropic effects [10].

While in this case it is hard to dispute the beneficial effect of inhaled marijuana at the prescribed dosage, it is possible that the effect would not last or that there would be a need to escalate the dosage. Therefore, this case report does not establish that the beneficial effects we observed would be maintained in the long run. However, the decrease of the number of potentially life threatening attacks from at least twice a month to nil and the effect of the drug on the patient's quality of life are encouraging.

More research into the exact mechanism of action of cannabis products in cases of idiopathic angioedema and on the modulation of the immune response in general is indicated.
